# Docetaxel-induced cognitive impairment in rats can be ameliorated by edaravone dexborneol: Evidence from the indicators of biological behavior and anisotropic fraction

**DOI:** 10.3389/fnins.2023.1167425

**Published:** 2023-04-03

**Authors:** Ping Liu, Hai Liu, Lijun Wei, Xun Shi, Wei Wang, Shengxiang Yan, Wenya Zhou, Jiangong Zhang, Suxia Han

**Affiliations:** ^1^Department of Radiation Oncology, The First Affiliated Hospital of Xi'an Jiaotong University, Xi'an, Shanxi, China; ^2^Department of Oncology, Heping Hospital Affiliated to Changzhi Medical College, Changzhi, Shanxi, China; ^3^Department of Urology Surgery, The People’s Hospital of Qijiang District, Chongqing, China; ^4^Department of Nuclear Medicine, The First People’s Hospital of Yancheng, The Fourth Affiliated Hospital of Nantong University, Yancheng, Jiangsu, China; ^5^Department of Science and Technology, Jiangsu Vocational College of Medicine, Yancheng, Jiangsu, China

**Keywords:** edaravone dexborneol, cognitive impairment, diffusion tensor imaging, hippocampus, fractional anisotropy

## Abstract

**Objective:**

This study aimed to investigate the effect of Edaravone Dexborneol (ED) on impaired learning and memory in docetaxel (DTX)-treated rats using cognitive behavior assessments and magnetic resonance diffusion tensor imaging (DTI).

**Materials and methods:**

In total, 24 male Sprague–Dawley rats were divided into control, low-dose DTX (L-DTX) model, and high-dose DTX(H-DTX) model groups, with eight rats in each group, numbered 1–8. The rats were intraperitoneally injected with 1.5 mL of either normal saline (control group), or 3 mg/kg and 6 mg/kg DTX (L-DTX and H-DTX groups, respectively), once a week for 4 weeks. The learning and memory abilities of each group were tested using a water maze. At the end of the water maze test, rats 1–4 in each group were treated with ED (3 mg/kg, 1 mL), and rats 5–8 were injected with an equal volume of normal saline once a day for 2 weeks. The learning and memory abilities of each group were evaluated again using the water maze test, and the image differences in the hippocampus of each group were analyzed using DTI.

**Results:**

(1) H-DTX group (32.33 ± 7.83) had the longest escape latency, followed by the L-DTX group (27.49 ± 7.32), and the Control group (24.52 ± 8.11) having the shortest, with the difference being statistically significant (*p* < 0.05). (2) Following ED treatment, compared to rats treated with normal saline, the escape latency of the L-DTX (12.00 ± 2.79 vs. 10.77 ± 3.97, *p* < 0.05), and the H-DTX (12.52 ± 3.69 vs. 9.11 ± 2.88, *p* < 0.05) rats were significantly shortened. The residence time in the target quadrant of H-DTX rats was significantly prolonged (40.49 ± 5.82 vs. 55.25 ± 6.78, *p* < 0.05). The CNS damage in the L-DTX rats was repaired to a certain extent during the interval between the two water maze tests (28.89 ± 7.92 vs. 12.00 ± 2.79, *p* < 0.05). (3) The fractional anisotropy (FA) value of DTI in the hippocampus of rats in the different groups showed variable trends. After treatment with ED, though the FA values of most areas in the hippocampus of rats in L-DTX and H-DTX groups were higher than before, they did not reach the normal level.

**Conclusion:**

ED can ameliorate the cognitive dysfunctions caused by DTX in rats by improving the learning and memory impairment, which is reflected in the recovery of biological behavior and DTI indicators of the hippocampus.

## 1. Introduction

Cancer is among the leading causes of death worldwide, with a continuous annual increase in the tumor detection rates. Though there have been many breakthroughs in cancer treatment strategies, chemotherapy still remains one of the mainstays with chemotherapeutic drugs being widely used in the clinical management of tumors. Docetaxel (DTX) is a semi-synthetic analog of paclitaxel belonging to the drug class taxanes, which are antimicrotubular agents, and act by interfering with mitosis in tumor cells. It is mostly used to treat lung, ovarian, and breast cancers (Onda et al., 2020; Tang et al., 2020; Wang J. et al., 2020; Wang S. et al., 2020). Studies found that the rate of paclitaxel-induced chemotherapy-related cognitive impairment during chemotherapy was up to 75%, also known as “chemotherapy brain,” and a small number of patients had cognitive impairment lasting for several months after chemotherapy (Alves et al., 2018; Das et al., 2020). Hippocampus plays a critical role in memory retrieval and spatial memory retention (Squire et al., 2010), and paclitaxel is known to induce hippocampal neuronal apoptosis (Li et al., 2017).

Edaravone is an antioxidant and a free-radical scavenger which has been shown to prevent neuronal death and brain edema (Abd. Aziz et al., 2020); and dexborneol can inhibit the expression of inflammatory cytokines, which may reduce apoptosis and necrosis (Xu et al., 2019). Edaravone dexborneol (ED) is a novel neuroprotective agent comprised of edaravone and dexborneol in 4:1ratio, which has been tested as a novel neuroprotective agent and was approved for listing in China on 30 July 2020. The American Heart/Stroke Association’s journal *Stroke* published the results of China’s ED phase III trial online (Xu et al., 2021). It was found that ED can protect hippocampal HT22 neurons, astrocytes, and vascular endothelial cells under stress, indicating that it can protect the entire neurovascular unit and inhibit neural apoptosis (Lee et al., 2010; Chen et al., 2022; Xu et al., 2022).

Diffusion tensor imaging (DTI) is an magnetic resonance imaging (MRI) technique based on diffusion-weighted imaging technology that can directly measure the microstructural integrity of brain white matter fiber tracts *in vivo*, and quantitatively display abnormal changes in the brain white matter (Lope-Piedrafita, 2018). In the central nervous system (CNS), water molecules usually diffuse parallel to the direction of the nerve fibers but rarely perpendicular to the long axis of the fiber, a phenomenon that is known as anisotropic diffusion. This degree of anisotropic diffusion is called fractional anisotropy (FA), which is the ratio of the anisotropic component of the diffusion tensor to the entire diffusion tensor that ranges from 0 to 1. When part of the brain tissue is degenerated or damaged, the FA value decreases (Dalboni da Rocha et al., 2020).

Chemotherapeutic drugs like DTX can cause damage to the hippocampus, whereas the newly marketed combination drug, ED, can protect and repair the damaged hippocampus. However, there is no research on the change in FA values in the hippocampus after injury, and after being treated for protection and repair. Therefore, this study aimed to explore the changes in FA values in the hippocampus in different states by establishing an animal model of rat hippocampal injury and using ED for intervention, to provide a theoretical basis for the appropriate clinical diagnosis and treatment of chemotherapy brain.

## 2. Materials and methods

### 2.1. Experimental animals

This study was approved by the ethics committee of our College (License No. SYLL-2021-800). In this study we used 24 pathogen-free male Sprague–Dawley rats (5 weeks old, 160–170 g), provided by the Nantong University (The License No. SCXK2019-0001). At the end of the experiment, rats were euthanized by spinal dislocation.

### 2.2. Model establishment

#### 2.2.1. Materials

The ANY-maze water maze test system was purchased from the Stoelting Company. DTX was provided by Chenxin Pharmaceutical Co., Ltd. (approval no.: GYZZ H20093647; specification, docetaxel injection: 0.5 mL:20 mg, docetaxel injection solvent: 1.5 mL). ED was provided by Xiansheng Pharmaceutical Co., Ltd. (approval number: GYZZ H20200007; 5 mL: edaravone [10 mg] and dexborneol [2.5 mg]).

#### 2.2.2. Animal grouping and DTX administration

**Figure 1:**
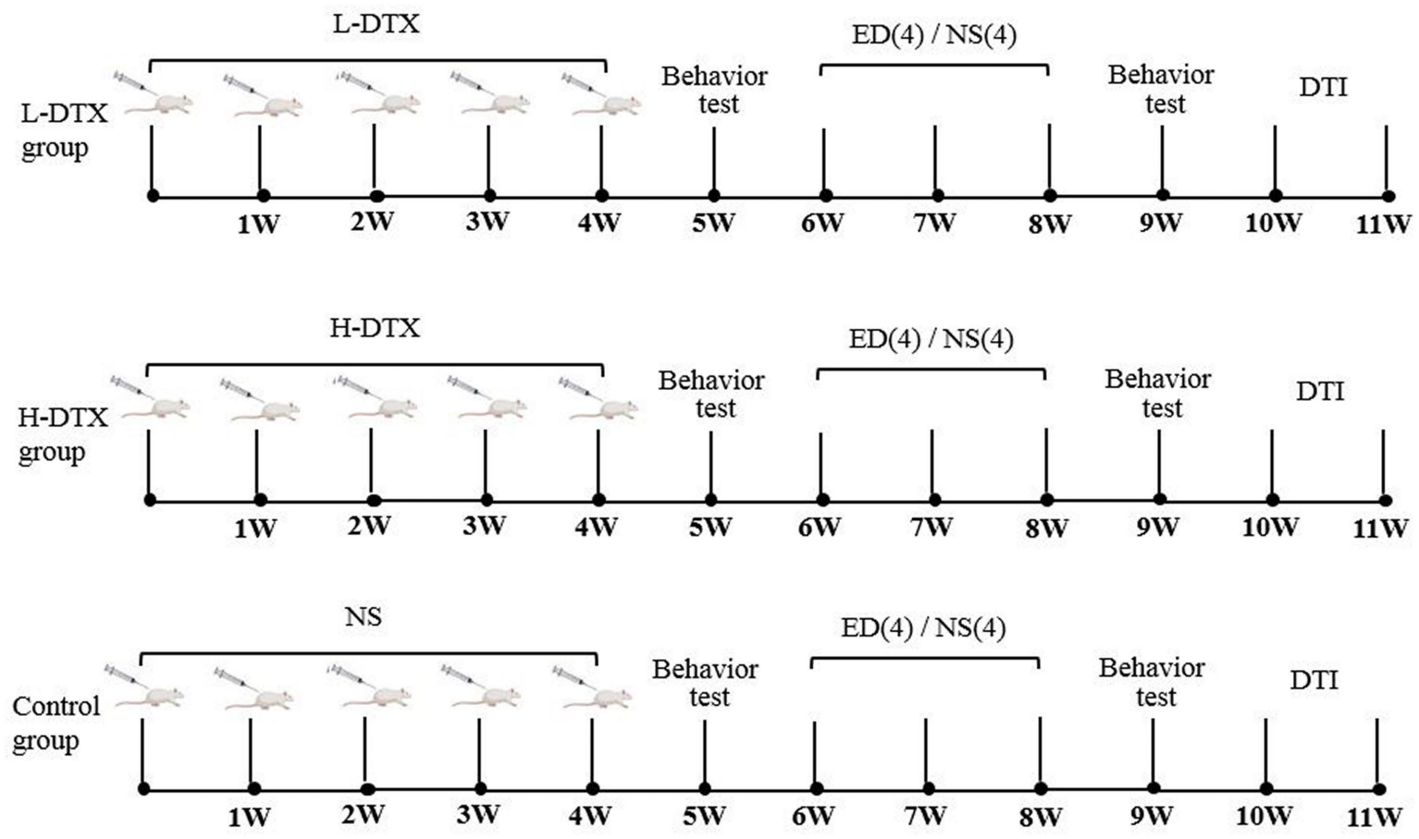
The experimental flow charts of L-DTX, H-DTX and control group rats, respectively. L-DTX，low-dose docetaxel; H-DTX, high-dose docetaxel; ED, edaravone dexborneol; NS, normal saline; DTI, Diffusion tensor imaging; W, week.

Before commencing the experiments, the ANY-maze water maze was used to test the rats’ learning and memory abilities. Those rats which could not swim smoothly were excluded. Finally, 24 selected rats were randomly divided into three groups, with eight rats (numbered: 1 to 8) in each of the following groups: low-dose DTX (L-DTX, 3 mg/kg), high-dose DTX (H-DTX, 6 mg/kg), and control (equal volume of normal saline). The animals were intraperitoneally injected with 1.5 mL DTX, once a week for four consecutive weeks (Figure 1).

### 2.3. The first water maze experiment

On the third day after the end of DTX injection regimen, a water maze test was conducted on rats using the ANY-maze to test their spatial learning and memory abilities. The specific parameters of the water maze were as follows: the diameter was 100 cm, water depth was 30 cm, water temperature was maintained at approximately 22°C, and pool was divided into four quadrants. The colorless circular platform (diameter, 9 cm) was located in the second quadrant, approximately 1–2 cm below the water surface. The background of the entire water maze was black, and white stickers of different shapes were attached to the middle of the inner wall of the four quadrants of the water maze as a reference for recognition and memory in rats. The experiment was divided into two stages over 6 days: the positioning cruise stage (Days 1–5), and the space search stage (Day 6). After rats entered the water, the ANY-maze tracking system was automatically run, and the timer was started with a time limit of 60 s. If the rats were on stage within 60 s, they were allowed to remain on the platform for 30 s. If the rats could not find the platform within 60 s, the rats were guided to the platform and kept there for 30 s (its escape latency was denoted as 60 s), during which the system automatically used cameras to track and record the swimming track of the rats in the pool, and special software calculated the escape latency for the rats to reach the platform. On day 6 of the space exploration experiment, the platform was removed, the rats were put into the water from the fourth quadrant (the diagonal quadrant of the platform), and the percentage of the rats’ stay time in the quadrant where the platform was located (the target quadrant), and the number of times they crossed the area where the platform was located were recorded and analyzed.

### 2.4. Edaravone dexborneol intervention analysis

#### 2.4.1. Animal grouping and administration

ED was administered on the third day of the first water maze test. Rats numbered 1–4 in each group were intraperitoneally injected with Edaravone dexborneol (ED; 3 mg/kg in 1 mL volume) once a day for 14 days in both, the L-DTX-ED and the H-DTX-ED groups. Rats numbered 5–8 in each group were injected with an equal volume of normal saline.

#### 2.4.2. The second water maze experiment

The second water maze experiment was started 1 day after the end of the ED treatment. The method used was the same as that described earlier.

### 2.5. Diffusion tensor imaging evaluations

#### 2.5.1. Magnetic resonance imaging in rats

MRI was performed with 9.4 T MR scanner (Bruker, Biospec) at Nanjing Drum Tower Hospital within 1 week of the biological behavior test (Figure 1). Rats were anesthetized with isoflurane (1.5 mL/min, 4% oxygen content), and the special head and neck coil was used for scanning with structural Magnetic resonance imaging (MRI) and diffusion tensor imaging (DTI).

High resolution T2WI was used for structural image of axial scanning: rapid acquisition with relaxation enhancement (RARE) sequence, TR = 5648.2 ms, TE = 33 ms, FOV = 25 mm × 25 mm, Matrix: 384 × 384, number of slices: 35, Slice thickness: 0.5 mm, resolution: 0.065 mm × 0.065 mm, Rare factor: 8. DTI for coronal imaging of rat brain was performed using a single shot spin echo planar imaging (EPI) sequence. Diffusion sensitivity gradient was 30 different directions, TR = 4,001 ms, TE = 22 ms, slice thickness 0.195 mm, FOV = 25 mm × 25 mm, acquisition matrix: 128 × 128, resolution: 0.195 mm × 0.195 mm, the number of slices: 47. The diffusion weighting coefficient (b value) were 0 and 1,000 s/mm^2^, respectively.

#### 2.5.2. Data processing

The original data was sorted into different groups and converted to the nii.gz format. The DTI files of each rat brain were preprocessed with FSL.^1^ Eddy correction, brain mask correction, rotate_bvecs correction, and the FA values of each hippocampal subregion were calculated with dtifit and SIGMA Rat Brain template [https://www.nitrc.org/; involving the brain regions of the fields CA1, CA2, and CA3 of the hippocampus, respectively; dentate gyrus (DG); ectorhinal (EC), lateral ectorhinal (LEC), and medial ectorhinal (MEC) cortex, respectively; subiculum, parasubiculum, and presubiculum in hippocampus, respectively].

### 2.6. Statistical analysis

The data are presented as the mean ± standard deviation. Escape latency data were obtained from the water maze localization navigation experiment using repeated measures of variance analysis. Other data were analyzed using one-way analysis of variance and the *t*-test. Statistical software (IBM Corp., SPSS 20.0) was used to perform statistical analysis. Statistical significance was set at *p* < 0.05.

## 3. Results

### 3.1. Effect of DTX on the learning and memory abilities of rats

#### 3.1.1. Positioning cruise phase

**Figure 2:**
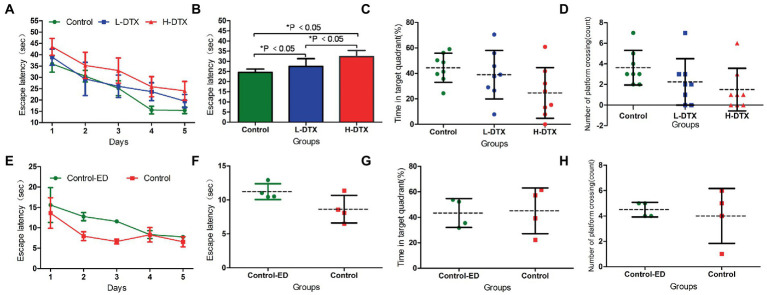
Panels **(A,B)** were the escape latency of Control, L-DTX and H-DTX groups. Panels **(C,D)** were the percentage of residence time in the target quadrant and the number of crossing the platform of the three groups of rats, respectively. Panels **(E,F)** were the escape latency of injected and non-injected ED rats in the control group. Panels **(G,H)** were the percentage of residence time in the target quadrant and the number of crossing the platform of injected and non-injected ED rats in the control group, respectively. L-DTX, low-dose docetaxel; H-DTX, high-dose docetaxel; ED, edaravone dexborneol.

The escape latency of the L-DTX and H-DTX groups was prolonged compared with that of the control group (*F* = 16.04,12.36, *p* < 0.05), and the escape latency of the H-DTX group was longer than that of the L-DTX group (*F* = 8.08, *p* < 0.05), as shown in Table 1 and Figures 2A,B. These findings indicate that DTX can cause damage to the CNS in rats, leading to decline in learning ability, with the decline being directly proportional to the dose of the DTX (see Supplementary material 1).

**Table 1:** Comparison of escape latency in three groups of rats.

Groups	*n*	1 day	2 days	3 days	4 days	5 days
Control	8	35.9 ± 10.21	30.50 ± 7.18	25.26 ± 9.31	15.56 ± 4.75	15.45 ± 4.14
L-DTX	8	38.93 ± 10.13	29.28 ± 20.79	26.04 ± 13.99	23.68 ± 11.13	19.51 ± 8.17
H-DTX	8	43.5 ± 10.56	35.26 ± 16.37	33.00 ± 15.75	25.84 ± 12.61	24.03 ± 11.67

L-DTX, low-dose docetaxel; H-DTX, high-dose docetaxel.

#### 3.1.2. Space exploration phase

After removing the platform, there was no differences in the percentage of stay time in the target quadrant and the number of times the rats crossed the platform between the control, L-DTX and H-DTX groups (*p* > 0.05; Table 2). However, it can be seen from the trend chart (Figures 2C,D) that the percentage of the target quadrant dwell-time and the number of platform crossings were the highest in the control group, followed by that in the L-DTX group, and the least in the H-DTX group.

**Table 2:** Comparison of spatial exploration ability among three groups of rats.

Groups	*n*	Time in target quadrant (%)	Number of platform crossing (count)
Control	8	44.38 ± 11.45	3.62 ± 1.69
L-DTX	8	38.98 ± 19.09	2.25 ± 2.25
H-DTX	8	24.62 ± 19.86	1.50 ± 2.07
F		2.81	2.28
P		0.083	0.126

L-DTX, low-dose docetaxel; H-DTX, high-dose docetaxel.

### 3.2. Horizontal analysis of ED efficacy among the different groups

#### 3.2.1. Effect of ED on the control rats

As shown in Tables 3, 4 and Figures 2E–H, the ED treatment had no effect on the escape latency and space exploration ability in the positioning navigation test of rats of the control group (*p* > 0.05; see Supplementary material 2).

**Table 3:** Effect of ED on escape latency of rats in control group, L-DTX group and H-DTX group.

Variables	*n*	1 day	2 days	3 days	4 days	5 days	F	*P*
Control-ED	4	15.61 ± 8.55	12.78 ± 2.02	11.60 ± 0.15	8.35 ± 1.95	7.78 ± 0.78	3.90	0.077
Control	4	13.63 ± 7.47	7.96 ± 2.18	6.66 ± 1.17	8.30 ± 3.54	6.58 ± 2.52		
L-DTX-ED	4	16.96 ± 9.29	11.73 ± 4.89	6.48 ± 1.60	10.14 ± 5.56	8.54 ± 4.79	4.61	0.007
L-DTX	4	16.39 ± 17.54	12.70 ± 10.18	11.43 ± 7.67	10.45 ± 9.61	9.05 ± 4.64		
H-DTX-ED	4	12.92 ± 4.18	11.50 ± 8.03	6.63 ± 0.71	7.36 ± 1.72	7.15 ± 0.83	5.74	0.002
H-DTX	4	18.61 ± 4.14	12.48 ± 4.60	12.33 ± 4.92	9.24 ± 2.59	9.94 ± 0.90		

ED, Edaravone Dexborneol; L-DTX, low-dose docetaxel; H-DTX, high-dose docetaxel.

**Table 4:** Effect of ED on spatial exploration ability of rats in Control group, L-DTX group and H-DTX group.

Variables	*n*	Time in target quadrant (%)	t	*P*	Number of platform crossing (count)	t	*P*
Control-ED	4	43.37 ± 11.32	0.16	0.876	4.50 ± 0.57	0.45	0.670
Control	4	45.08 ± 17.97			4.00 ± 2.16		
L-DTX-ED	4	49.96 ± 15.08	0.38	0.715	4.75 ± 2.62	0.14	0.893
L-DTX	4	45.54 ± 17.44			4.50 ± 2.38		
H-DTX-ED	4	55.25 ± 6.78	3.30	0.016	6.00 ± 0.82	0.12	0.903
H-DTX	4	40.49 ± 5.82			6.25 ± 3.86		

ED, Edaravone Dexborneol; L-DTX, low-dose docetaxel; H-DTX, high-dose docetaxel.

#### 3.2.2. Effect of ED on L-DTX rats

**Figure 3:**
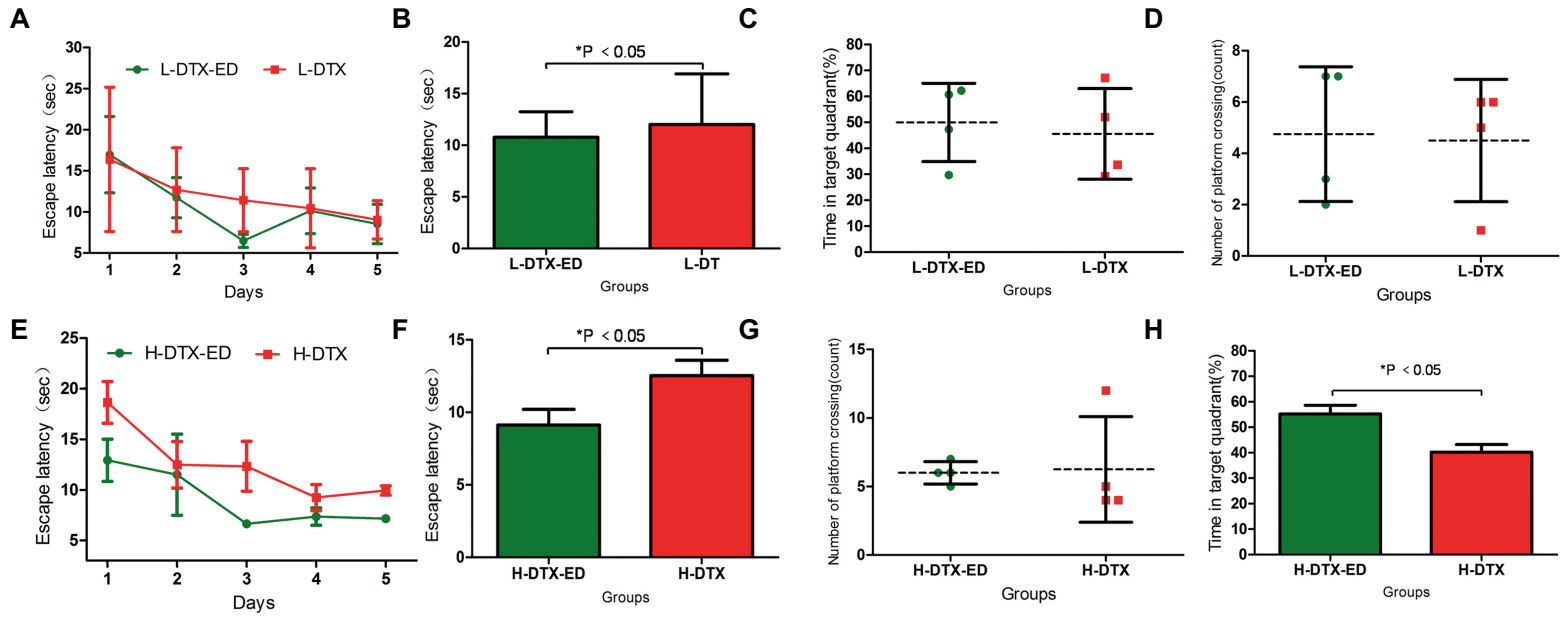
Panels **(A,B)** were the escape latency of L-DTX group rats injected with and without ED; Panels **(C,D)** were the percentage of residence time in the target quadrant and the number of crossing the platform of L-DTX group rats injected with and without ED, respectively. Panels **(E,F)** were the escape latency of H-DTX group rats injected with ED and those not injected with ED; Panels **(G,H)** were the percentage of residence time in the target quadrant and the number of crossing the platform of H-DTX group rats injected with and without ED, respectively. L-DTX, low-dose docetaxel; H-DTX, high-dose docetaxel; ED, edaravone dexborneol.

The escape latency of the positioning navigation test was shortened in the L-DTX-ED group compared to the L-DTX group (*p* < 0.05; Table 3, Figures 3A,B). Although there was no difference in the spatial exploration ability between the two groups (*p* > 0.05; Table 4), the residence time in the target quadrant and the number of crossings on the platform of the L-DTX-ED group showed an increasing trend (Figures 3C,D). These results suggest that ED has a protective effect against a low dose of DTX-induced CNS injury in rats.

#### 3.2.3. Effect of ED on H-DTX rats

ED injection in H-DTX rats led to shortening of the escape latency (*p* < 0.05, Table 3; Figures 3E,F). Regarding space exploration ability, the percentage of residence time in the target quadrant of H-DTX-ED rats increased (*p* < 0.05; Table 4; Figure 3G), and the number of crossings on the platform also increased (Table 4; Figure 3H). These results indicate that ED also was protective against CNS damage caused by high dose of DTX as well.

### 3.3. Longitudinal analysis of ED efficacy among the different groups

#### 3.3.1. Protective effect of ED on L-DTX rats

##### 3.3.1.1. Recovery of L-DTX rats between the two water maze tests

**Figure 4:**
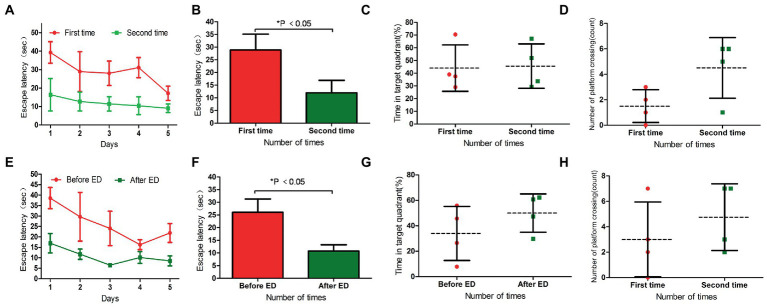
Panels **(A,B)** were the escape latency of two water maze experiments in L-DTX group. Panels **(C,D)** were the percentage of residence time in the target quadrant and the number of crossing the platform in the two water maze experiments of L-DTX group, respectively. Panels **(E,F)** were the escape latency of L-DTX group rats before and after ED injection; Panels **(G,H)** were the percentage of residence time in the target quadrant and the number of crossing the platform before and after ED injection of L-DTX group. L-DTX, low-dose docetaxel; ED, edaravone dexborneol.

The escape latency of L-DTX rats was shortened in the second water maze navigation test compared with the first water maze navigation test (*p* < 0.05; Table 5; Figures 4A,B). Although there was no statistical difference in space exploration ability (*p* > 0.05; Table 6), there was an increasing trend (Figures 4C,D). These results indicate that the CNS damage caused by L-DTX in rats was repaired to a certain extent during the interval between the two water maze tests.

**Table 5:** Comparison of two escape latency of rats in L-DTX group and H-DTX group.

Days	L-DTX group (*n* = 4)				H-DTX group (*n* = 4)			
	First time	Second time	F	*P*	First time	Second time	F	*P*
1 day	39.27 ± 11.66	16.39 ± 17.54	6.14	0.002	47.55 ± 11.45	18.64 ± 4.13	2.53	0.067
2 days	28.92 ± 21.53	12.70 ± 10.18			42.87 ± 19.57	12.48 ± 4.59		
3 days	28.02 ± 13.22	11.43 ± 7.67			33.55 ± 18.46	12.33 ± 4.92		
4 days	31.08 ± 10.98	10.45 ± 9.61			30.07 ± 11.17	9.24 ± 2.59		
5 days	17.15 ± 7.74	9.05 ± 4.64			31.02 ± 10.92	9.34 ± 0.90		

L-DTX, low-dose docetaxel; H-DTX, high-dose docetaxel.

**Table 6:** Comparison of two spatial exploration abilities of rats in L-DTX group and H-DTX group.

Indexes	L-DTX group (*n* = 4)				H-DTX group (*n* = 4)			
	First time	Second time	t	*P*	First time	Second time	t	*P*
Time in target quadrant (%)	44.00 ± 18.21	45.54 ± 17.44	0.12	0.910	21.00 ± 27.28	40.49 ± 5.82	1.38	0.217
Number of platform crossing (count)	1.50 ± 1.29	4.50 ± 2.38	2.22	0.082	1.00 ± 1.41	6.25 ± 3.86	2.55	0.066

L-DTX, low-dose docetaxel; H-DTX, high-dose docetaxel.

##### 3.3.1.2. Neuroprotective effect of ED in L-DTX rats

After the injection of ED, the escape latency of L-DTX rats was significantly shortened (*p* < 0.05; Table 7; Figures 4E,F). Although there was no difference in space exploration ability (*p* > 0.05; Table 8; Figures 4G,H), there was an increasing trend.

**Table 7:** Comparison of escape latency between L-DTX group and H-DTX group before and after ED injection.

Days	L-DTX group (*n* = 4)				H-DTX group (*n* = 4)			
	Before ED	After ED	F	*P*	Before ED	After ED	F	*P*
1 day	38.57 ± 10.15	16.96 ± 9.29	4.38	0.008	39.45 ± 9.24	12.92 ± 4.18	3.60	0.020
2 days	29.63 ± 23.33	11.73 ± 4.89			27.65 ± 9.36	11.50 ± 8.03		
3 days	24.05 ± 16.48	6.48 ± 1.60			32.45 ± 15.41	6.63 ± 0.71		
4 days	16.27 ± 4.76	10.14 ± 5.56			21.60 ± 14.07	7.36 ± 1.72		
5 days	21.88 ± 9.00	8.54 ± 4.79			17.02 ± 8.24	7.15 ± 0.83		

ED, Edaravone Dexborneol; L-DTX, low-dose docetaxel; H-DTX, high-dose docetaxel.

**Table 8:** Comparison of spatial exploration ability of L-DTX group and H-DTX group rats before and after ED injection.

Indexes	L-DTX group (*n* = 4)				H-DTX group (*n* = 4)			
	Before ED	After ED	t	*P*	Before ED	After ED	t	*P*
Time in target quadrant (%)	33.95 ± 21.24	49.96 ± 15.08	1.23	0.265	28.25 ± 11.85	55.25 ± 6.78	3.95	0.008
Number of platform crossing (count)	3.00 ± 2.94	4.75 ± 2.62	0.89	0.410	2.00 ± 2.78	6.00 ± 0.82	2.83	0.055

ED, Edaravone Dexborneol; L-DTX, low-dose docetaxel; H-DTX, high-dose docetaxel.

#### 3.3.2. Protective effect of ED on H-DTX rats

##### 3.3.2.1. Recovery of H-DTX rats between the two water maze tests

**Figure 5:**
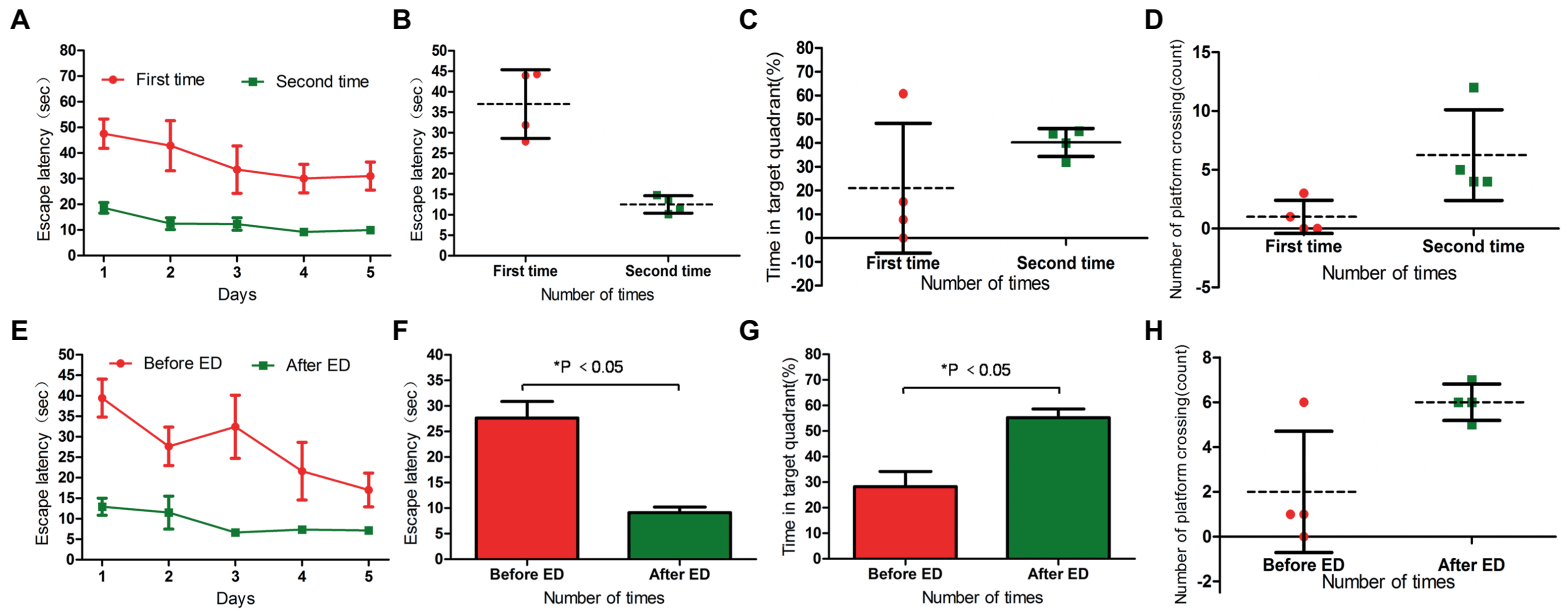
Panels **(A,B)** were the escape latency of two water maze experiments in H-DTX group. Panels **(C,D)** are the percentage of residence time in the target quadrant and the number of crossing the platform in the two water maze experiments of H-DTX group, respectively. Panels **(E,F)** were the escape latency of H-DTX group rats before and after ED injection; Panels **(G,H)** were the percentage of residence time in the target quadrant and the number of crossing the platform before and after ED injection of H-DTX group. H-DTX, high-dose docetaxel; ED, edaravone dexborneol.

In the two water maze experiments, H-DTX rats showed no difference in escape latency and space exploration ability in the positioning navigation experiment (*p* > 0.05; Tables 5, 6; Figures 5A–D). These results indicate that the interval between the two water maze experiments had no significant effect on the recovery of CNS injury induced by H-DTX in rats.

##### 3.3.2.2. Neuroprotective effect of ED in H-DTX rats

The escape latency of H-DTX rats was significantly shortened after ED injection (*p* < 0.05; Table 7; Figures 5E,F). The percentage of residence time in the target quadrant increased (*p* < 0.05; Table 8; Figure 5G), and the number of times the platform was crossed also increased (Figure 5H). These results indicate that ED has a protective effect on CNS injury induced by H-DTX in rats.

### 3.4. Trend analysis of biological behavior and neuroimage parameters in rats

**Figure 6:**
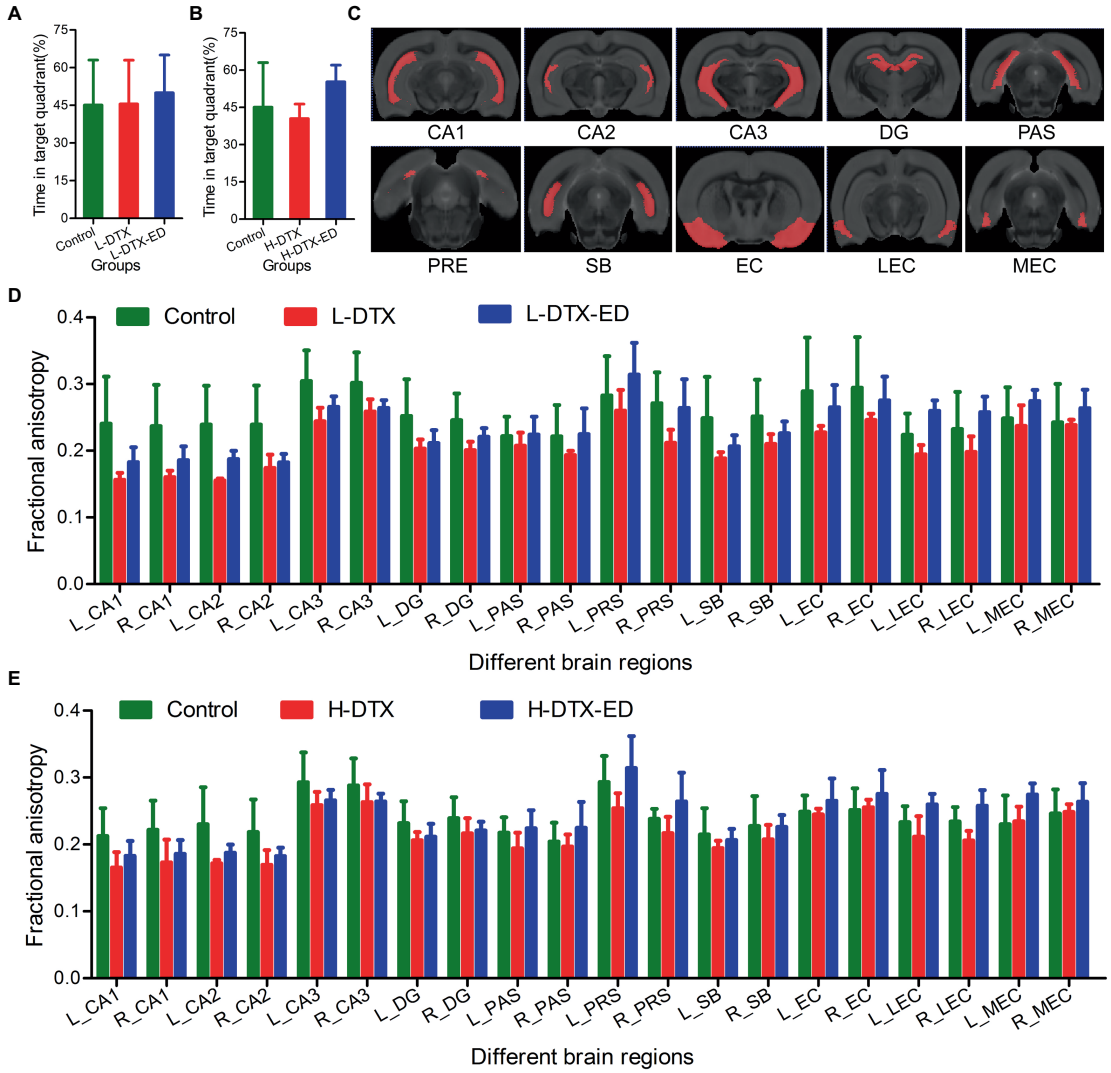
Panels **(A,D)** were the percentage of residence time in target quadrant and the FA value in DTI images of rats in Control group, L-DTX group and L-DTX-ED group, respectively. Panel **(C)** was the model map of 10 pairs of brain regions in DTI images of hippocampus. Panels **(B,E)** were the percentage of residence time in the target quadrant of the Control group, H-DTX and H-DTX-ED group and the FA value in the DTI image, respectively. L-DTX, low-dose docetaxel; H-DTX, high-dose docetaxel; ED, edaravone dexborneol; DTI, diffusion tensor imaging; FA, fractional anisotropy; PAS, Parasubiculum; PRE, Presubiculum; SB, Subiculum.

The DTI quantitative parameter FA in the hippocampal region of rats of different groups showed different trends. After treatment with ED, the FA values of most areas in hippocampus of rats in L-DTX and H-DTX groups were higher than before, but they did not reach the normal level, as shown in Figure 6 (see Supplementary materials 3, 4).

## 4. Discussion

Cancer survivors frequently suffer cognitive disturbances following chemotherapy, in particular, memory loss associated with hippocampal dysfunction. The cognitive dysfunctions in these patients seriously affects their quality of life by reducing memory, attention, and response speed (Hodgson et al., 2013). DTX, a taxane chemotherapeutic agent with high anti-tumor activity, leads to microtubule dysfunction and cell death through the inhibition of microtubule dynamics. Previous studies have shown that the drug class taxanes can inhibit the activity of hippocampal cells and induce hippocampal nerve apoptosis (Li et al., 2017; Tang et al., 2022).

The hippocampus is an important part of the limbic system and plays an important role in learning, memory, emotional responses, and pathophysiological changes in CNS diseases.

Radiolabeled paclitaxel has been detected in the brain using positron emission computed tomography techniques, showing that it can cross the blood–brain barrier and enter the brain. This neurotoxicity may be related to the dose used (Gangloff et al., 2005). Herein, the escape latency of the L-DTX and H-DTX groups was prolonged during the positioning cruise stage compared with the control group, and the escape latency of the H-DTX group was longer than that of the L-DTX group; the differences were statistically significant. In the space exploration stage, the percentage of residence time in the target quadrant and the number of platform crossings increased successively in the H-DTX, L-DTX, and control groups. This result also indirectly confirmed some of the aforementioned views: (1) DTX can cause CNS damage in rats and lead to decreased learning ability; (2) the higher the dose, the more severe the degree of decreased learning ability in rats. Gamma-aminobutyric acid (GABA) is an inhibitory neurotransmitter in the CNS and plays important physiological roles mainly through interaction with its specific receptors. Autoradiography and immune markers show that GABA_B_ receptors are widely distributed in the CNS of mammals (such as the thalamus, hypothalamus, hippocampus, and cerebral cortex) and play a key role in cognitive learning (Smid et al., 2001). Masocha and Parvathy (2016) suggested that GABA_B_ may be an important receptor in paclitaxel-induced neuropathic pain and cognitive dysfunction. The endoplasmic reticulum is an important organelle for protein synthesis, folding, and secretion in eukaryotic cells as well as an important site for Ca^2+^ storage. When cells are stimulated, misfolded proteins accumulate to a certain extent and the Ca^2+^ balance is disturbed, resulting in endoplasmic reticulum stress. Endoplasmic reticulum stress of the CNS has been shown to be closely related to cognitive dysfunction caused by human nervous system diseases, and paclitaxel can induce neurotoxicity by activating endoplasmic reticulum stress (Janczar et al., 2017; Logue et al., 2018; Chen et al., 2019; Lin et al., 2020).

In the cross-sectional study of this experiment, it was found that after the injection of ED in the L-DTX group, the escape latency of the positioning navigation test was shortened, and the residence time in the target quadrant and number of crossings were increased. In case of H-DTX rats, treatment with ED led to shortening of the escape latency and enhancement of their space exploration ability. This study’s result indicate that ED has a protective and repairing effect on CNS damage caused by both, low and high doses of DTX in rats. ED contains two components with multiple effects, such as scavenging free radicals, anti-inflammation, and protection of the blood–brain barrier, covering a broad spectrum of mechanism of post-ischemic nerve damage. Many studies have shown that edaravone can reduce inflammatory and oxidative stress damage by inhibiting inflammatory activation, promoting antioxidant pathways, and antagonizing and restoring apoptosis-related regulatory factors, through various signaling pathways to play a neuroprotective role in brain tissue, including inhibition of neural function defects, cell apoptosis, and structural damage (Wu et al., 2014; Zhang et al., 2019; Guo et al., 2020). The advantage of combining dexborneol with edaravone is that it can effectively inhibit various inflammatory cytokines and chemokines in the inflammatory cascade reaction, reduce the mixed attack of oxygen-free radicals and inflammatory factors on neurovascular units, and further strengthen the protective effect on neurons. The results of an ED phase III trial became strong evidence and proved that ED had a better protective effect on nerves than edaravone alone (Xu et al., 2021).

Another finding of this study is that less damaging cognitive dysfunction can recover to varying degrees within a certain period. The longitudinal research results of this experiment showed that the learning and memory impairment of rats induced by low DTX dosage recovered to a certain extent during the time interval between the two water maze tests, but there was no obvious recovery effect on rats with learning and memory impairment induced by high dose of DTX. Earlier studies have also confirmed that, among the patients with breast cancer with mild to moderate cognitive impairment in the course of chemotherapy, 35% of patients have persistent symptoms for months to years after termination of the treatment (Janelsins et al., 2011). Combined with this study’s findings, it is suggested that the dose of chemotherapy drugs affects the severity and duration of cognitive dysfunction. This dose-dependent neurotoxicity can significantly reduce patients’ quality of life; therefore, special attention should be paid for preventing excessive chemotherapeutic doses in the clinical setting.

As a brain research tool, magnetic resonance DTI provides brain microstructure images with higher sensitivity, and more detailed comparison for the diagnosis of brain diseases than other imaging modalities, thus having clear advantages over other diagnostic methods. In this study, DTI quantitative analysis was used to study the degree of damage caused by DTX on cognitive dysfunction and the neuroprotective effect of ED intervention. The results showed that in the 10 pairs of brain regions, FA values of the hippocampus were generally higher in the control group than in the other two groups; those in which regions were protected by ED were in the middle, whereas those in which regions were not protected by ED were generally lower. This indicated that after treatment with ED, the FA values of most areas in hippocampus of rats in L-DTX and H-DTX groups were higher than before, but they did not reach the normal level. The larger the FA value, the more regular the dispersion movement of water molecules, the more regular the organizational arrangement, and better the integrity. When the integrity of white matter fibers is damaged, the dispersion barrier is damaged, resulting in weakening of the dispersion ability of water molecules along the direction of white matter fiber bundles, together with enhancement of the dispersion ability to the periphery, and consequently, FA value is decreased. Many studies have also shown that the FA value of the hippocampus is generally reduced in patients with mild cognitive impairment (Zhao et al., 2014; Allen et al., 2019). The fornix is composed of output fibers of the hippocampus and is an important structure that maintains its normal function. Some studies found that the FA value of the fornix in patients with mild cognitive impairment was also reduced, and this index was correlated with the memory score (Mielke et al., 2012; Nowrangi et al., 2013). In other words, the fornix in patients with mild cognitive impairment had damaged integrity of the white matter of the brain, with axonal degeneration and demyelination changes, and the extent of the lesion was related to the degree of memory decline.

This study has some limitations. First, because the number of rats treated with DTX becomes less after being divided into more detailed treatment group and untreated group, it will likely to have some impact on the results. Second, we only uses the subregions of hippocampus as the indicators to analyze various abnormal FA values. Since brain activity does not occur in isolated regions of the brain, we plan to optimize experimental grouping and number of animals, and further explore ED-related protection mechanisms by using whole brain region and network analysis in the future.

In conclusion, the result of this study indicates that Docetaxel treatment can cause cognitive dysfunction of rats and ED can improve the chemotherapy-related impairment mainly reflected in the recovery of biological behavior and DTI indicators of the hippocampus.

## Data availability statement

The original contributions presented in the study are included in the article/Supplementary material, further inquiries can be directed to the corresponding authors.

## Ethics statement

The animal study was reviewed and approved by the Ethics Committee of Jiangsu Vocational College of Medicine.

## Author contributions

PL acquired and analyzed data, drafted the manuscript, and revised it. HL, LW, XS, WW, SY, and WZ analyzed and explained the data. SH and JZ designed and revised the manuscript for extremely important intellectual content. All authors contributed to the article and approved the submitted version.

## Funding

This study was supported by funding from 2021 Yancheng Medical Science and Technology Development Project Grants-Yancheng Health Planning [2021] No. 47 (YK2021017), Institute-Level Scientific Research Fund of Peace Hospital Affiliated to Changzhi Medical College (HPYJ202220), and The Health Commission of Shanxi Province (no. 2021008).

## Conflict of interest

The authors declare that the research was conducted in the absence of any commercial or financial relationships that could be construed as a potential conflict of interest.

## Publisher’s note

All claims expressed in this article are solely those of the authors and do not necessarily represent those of their affiliated organizations, or those of the publisher, the editors and the reviewers. Any product that may be evaluated in this article, or claim that may be made by its manufacturer, is not guaranteed or endorsed by the publisher.

## Footnotes

^1^https://fsl.fmrib.ox.ac.uk/fsl/fslwiki/
